# Effects of diet before endurance exercise on hepcidin response in young untrained females

**DOI:** 10.20463/jenb.2018.0030

**Published:** 2018-12-31

**Authors:** Nanako Hayashi, Aya Ishibashi, Kazushige Goto

**Affiliations:** 1 Graduate School of Sports and Health Science, Ritsumeikan University, Shiga Japan; 2 Department of Sports Science, Japan Institute of Sports Science, Tokyo Japan

**Keywords:** Iron metabolism, Interleukin-6, Carbohydrate

## Abstract

**[Purpose]:**

We examined the effects of diet before endurance exercise on hepcidin response in young untrained females.

**[Methods]:**

Ten young untrained females [age: 20.6 ± 0.8 y, height: 157.5 ± 1.0 cm, weight: 54.4 ± 1.5 kg, and maximal oxygen uptake (VO_2max_): 35.9 ± 1.1 mL/kg/min] were involved in two experimental conditions with a crossover design. The two conditions were separated by approximately 1 month, and each condition was performed during the follicular phase. Subjects completed 60 min of pedaling at 65% of VO_2max_ after consuming a meal (FED) or not consuming a meal (CON). Blood samples were collected before, immediately after, and 3 h after exercise.

**[Results]:**

Serum ferritin levels before exercise did not differ between the two conditions (*P *> 0.05). Blood glucose and lactate levels were significantly elevated immediately after exercise only under the FED condition (*P* < 0.05). Serum iron levels were significantly elevated after exercise under both conditions. However, the plasma interleukin-6 and serum hepcidin levels were not significantly different 3 h after exercise under either condition (*P* > 0.05).

**[Conclusion]:**

Consuming a meal before endurance exercise at moderate intensity did not affect exercise-induced hepcidin elevation in young untrained females.

## INTRODUCTION

Exercise-induced iron deficiency is a prevalent disorder among female endurance athletes^1^. It is believed to result from insufficient iron intake from daily meals, destruction of red blood cells (augmented hemolysis), sweating, and gastrointestinal bleeding^2-5^. Studies on hepcidin (an iron-regulating hormone) have been growing as a potential factor associated with iron deficiency in endurance athletes^6-7^.

Hepcidin is a master regulator of iron metabolism^8^. It triggers the degradation of ferroportin (an iron exporter protein) in the intestine and on the surfaces of macrophages^9,10^, thereby reducing dietary iron absorption and the release of iron from macrophages (impaired iron recycling from damaged erythrocytes). Therefore, an increase in the hepcidin level compromises iron availability^11^. Several studies have demonstrated that exercise acutely increases serum hepcidin levels during the post-exercise period^7,12-14^. Furthermore, exercise-induced increases in interleukin-6 (IL-6) are believed to stimulate the subsequent increase in hepcidin. Robson-Ansley et al. (2011) reported a significant correlation between IL-6 and hepcidin levels after 2 h of running at 60% of maximal oxygen uptake ($\dot{V}$O_2max_)^15^. An exercise-induced increase in the hepcidin level is generally observed following an increase in IL-6, with a peak at approximately 3 h after exercise has been completed^12,13^. A primary factor that increases IL-6 production with prolonged exercise is the degradation of muscle glycogen^16,17^. In addition, the utilization of exercise-induced muscle glycogen is influenced by dietary intake, with carbohydrate ingestion before or during exercise attenuating the use of muscle glycogen (i.e., sparing effects of muscle glycogen). Moreover, exercise in the morning may augment the exercise-induced elevation of IL-6 with a subsequent increase in serum hepcidin levels because glycogen content is lower in the morning following overnight fasting. Therefore, it is possible that exercise following overnight fasting (and skipping breakfast) promotes an exercise-induced increase in serum hepcidin levels. 

Several studies have focused on the effects of ingesting carbohydrates on the hepcidin response. Badenhorst et al. (2015) reported that the timing of carbohydrate ingestion after exercise (2 h vs. 4 h after exercise completion) did not affect exercise-induced increases in IL-6 or hepcidin^18^. Sim et al. (2012) also showed that ingesting carbohydrate (every 20 min) did not attenuate the IL-6 or hepcidin response during 90 min of running at 75% of $\dot{V}$O_2max_^19^. In contrast, Badenhorst et al. (2015) manipulated muscle glycogen content owing to exercise (to deplete muscle glycogen) and altered carbohydrate intake during the post-exercise period^20^. The resting hepcidin levels were significantly elevated on the following morning when carbohydrate ingestion was restricted after exercise. However, in this experiment, no effects of carbohydrate restriction on post-exercise hepcidin elevation were evident^20^. Carbohydrate ingestion before endurance exercise may attenuate hepcidin elevation because an elevated blood glucose level facilitates blood glucose utilization during exercise, leading to the preservation of muscle glycogen. Furthermore, exercise-induced hepcidin elevation has not been fully elucidated in untrained females. 

Therefore, we investigated the effects of consuming a meal (containing enough carbohydrates) before prolonged exercise on the hepcidin response in young, untrained, female subjects. 

We hypothesized that eating before exercise would attenuate exercise-induced elevations in IL-6 and hepcidin levels.

## METHODS

### Subjects

Ten young untrained female subjects participated in the present study (mean ± standard error of the mean [SE]: age: 20.6 ± 0.8 years, height: 157.5 ± 1.0 cm, body weight: 54.4 ± 1.5 kg, percent body fat: 27.5 ± 1.1%, and $\dot{V}$O_2max_: 35.9 ± 1.1 mL/kg/min). None of the subjects had an irregular menstrual cycle, and the subjective symptom of anemia was taken into account. Each subject was informed of the purpose of the study, the experimental procedures, and the possible risks associated with the study. All subjects provided written informed consent. The present study was approved by the Ethical Committee for Human Experiments at Ritsumeikan University (BKC-IRB-2015-039-1), in accordance with the Declaration of Helsinki. 

### Experimental overview

Subjects visited the laboratory three times throughout the experimental period. On the first visit, they conducted a graded pedaling test to determine their $\dot{V}$O_2max_ and to calculate the workload during exercise for the main trials. The two main trials, consisting of exercise after consuming a meal (FED) or without consuming a meal (CON), were performed on the second and third visits. All subjects conducted a 60 min pedaling exercise at 65% of $\dot{V}$O_2max_ under either fed (FED) or fasting (CON) conditions. The order of these two conditions was randomized. An approximate 1-mo period between experimental visits was followed to ensure that the two conditions were completed during the follicular phase (7–11 d from the start of menstruation)^21,22^. 

### FED and CON conditions

Overview of the experiment on the testing day was presented in Table 1. Subjects arrived at the laboratory in the morning (8:00) following an overnight fast (10 h of fasting). After resting for 15 min, a baseline blood sample was collected from an antecubital vein. The subjects undertook the FED condition with consuming breakfast (509 kcal; 83% from carbohydrate, 8% from protein, and 9% from fat). They were requested to complete eating within 10 min. In the CON condition, the subjects continued to rest without consuming breakfast. At 30 min after starting to eat breakfast (in FED) or an identical period of rest (in CON), a 60 min pedaling exercise commenced^23,24^. Further blood samples were collected immediately after exercise and 3 h after exercise. Water intake was allowed during exercise. The amount of water consumed was recorded during the first condition and subjects consumed the same amount during the second condition. 

**Table 1.** Average heart rate and rating of percieved exertion during exercise

	FED	CON
HR (bpm)	165 ± 4	161 ± 4
RPE		
15 min	4.7 ± 0.5	4.1 ± 0.3
30 min	5.9 ± 0.3	5.7 ± 0.4
45 min	5.8 ± 0.4	5.8 ± 0.5
60 min	6.7 ± 0.4	6.2 ± 0.4

Values are means ± SE. HR; heart rate, RPE; ratings of perceived exertion

FED; exercise after consuming a meal, CON; exercise without consuming a meal

### Maximal oxygen uptake 

The graded pedaling test to determine $\dot{V}$O_2max_ was conducted on a bicycle ergometer (Aerobike 75XLIII, Konami Sports Life Co., Ltd, Kanagawa, Japan). The initial load was 30 W with increments of 30 W every 2 min until 90 W was reached. Subsequently, the load was further increased by 20 W until exhaustion. Respiratory gases were collected throughout the exercise with an automatic gas analyzer (AE300S; Minato Medical Science, Tokyo, Japan). The sample during exercise was analyzed to determine the $\dot{V}$O_2max_, and the workload at 65% of $\dot{V}$O_2max_ was calculated individually.

### Energy expenditure

Respiratory gases were collected at 25–30 min and 55–60 min using an automatic gas analyzer (AE300S; Minato Medical Science, Tokyo, Japan) to evaluate $\dot{V}$O_2_, $\dot{V}$CO_2_ $\dot{V}$E, and the respiratory exchange ratio (RER). Energy expenditure was calculated as 3.9 × $\dot{V}$O_2_ + 1.1 × $\dot{V}$CO_2_^25^.

### Ratings of perceived exertion and heart rate 

Ratings of perceived exertion (RPE) and heart rate (HR) were evaluated during the 60 min of exercise. RPE was recorded every 15 min during exercise using a modified Borg scale (0–10)^26^. HR was evaluated every 1 min throughout the 60 min of exercise using a wireless HR monitor (RCX5; Polar, Tokyo, Japan). 

### Blood sampling and analyses

Blood samples were collected from an antecubital vein before exercise (before the start of the exercise period), immediately after exercise, and 3 h after exercise. The samples were centrifuged (3,000 rpm, 4°C), and serum and plasma samples were stored at -80°C until subsequent analyses. Serum ferritin, iron, and hepcidin levels were measured. Ferritin and iron levels were measured at a clinical laboratory (SRL Inc., Tokyo, Japan), and hepcidin levels were analyzed using an enzyme-linked immunosorbent assay (ELISA) kit (R&D Systems, Minneapolis, MN, USA). Plasma IL-6 levels were measured using an ELISA kit (Human IL-6 Quantikine HS; R&D Systems). Glucose and lactate levels were measured immediately after the collection of blood using a glucose analyzer (Freestyle, Nipro Co., Osaka, Japan) and a lactate analyzer (Lactate Pro; Arkray Co., Kyoto, Japan), respectively.

### Statistical analyses

All data are presented as mean ± SE. A repeated-measures analysis of variance was used to analyze the interaction (condition × time) and main effects. A post hoc test (Tukey-Kramer test) was conducted when a significant interaction or main effects was found. *P* values < 0.05 were considered statistically significant.

## RESULTS

### HR and RPE 

Average HR and RPE during exercise are presented in Table 1. No significant differences in HR during exercise were observed in HR during exercise between the FED and CON conditions (*P* > 0.05). The RPE scores during exercise significantly increased as exercise progressed (main effect for time,* P* = 0.03). However, no differences were observed at any time point between the FED and CON conditions. (main effect for condition, *P* = 0.325)

### Energy expenditure and RER

Energy expenditure and RER during exercise are presented in Table 2. Energy expenditure during endurance exercise was not significantly different between the FED and CON conditions. RER showed a significant decrease with the progress of exercise (main effect for time, *P* = 0.002). RER during endurance exercise was higher in the FED condition than in the CON condition, and a significant difference between the conditions was observed at the 55–60 min of exercise (main effect for condition, *P* < 0.01). 

**Table 2.** Energy expenditure and respiratory exchange ratio during exercise

	FED	CON
EE (kcal)	431 ± 26	421 ± 25
RER		
30 min	0.96 ± 0.01	0.93 ± 0.01
60 min	0.93 ± 0.01^*^^†^	0.89 ± 0.01^*^

Values are means ± SE ^†^ ; P < 0.05 (FED vs. CON), * ; P < 0.05 vs. 30 min

EE; energy expenditure, RER; respiratory exchange ratio

FED; exercise after consuming a meal, CON; exercise without consuming a meal

### Blood variables

Fig. 2 presents blood glucose and lactate levels. Blood glucose levels significantly increased immediately after exercise in the FED condition, whereas no change was observed in the CON condition. In addition, the FED condition had a significantly higher blood glucose level than that of the CON condition immediately after exercise (FED: 114 ± 5 mg/dL, CON: 92 ± 3 mg/dL, *P* < 0.01). Blood lactate levels significantly increased immediately after exercise in the FED and CON conditions (main effect for time, *P* < 0.01), and these were significantly higher in the FED condition than that in the CON condition [FED: 4.2 ± 0.6 mmol/L, CON: 3.1 ± 0.6 mmol/L, interaction (condition × time), *P* < 0.01]. Baseline serum ferritin levels did not significantly differ between the conditions (*P *> 0.05).

**Figure 1.**
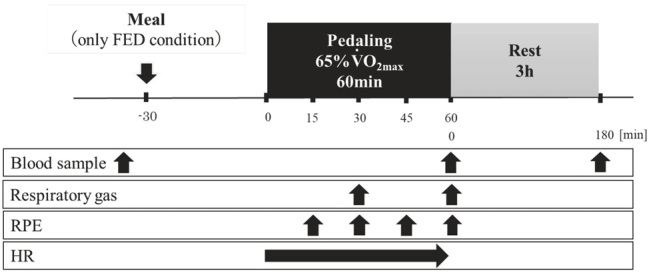
Overview of the experiment on the testing day.

**Figure 2.**
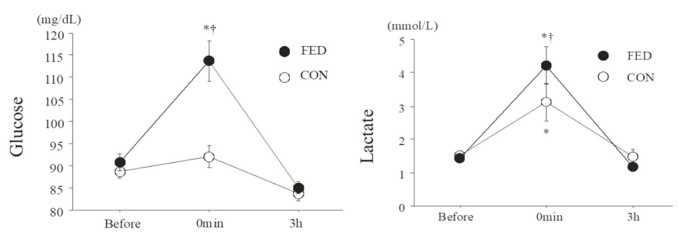
Blood glucose and lactate levels before and after exercise. Values are means ± SE. *; P < 0.05 vs. before †; P < 0.05 (FED vs. CON).

**Figure 3.**
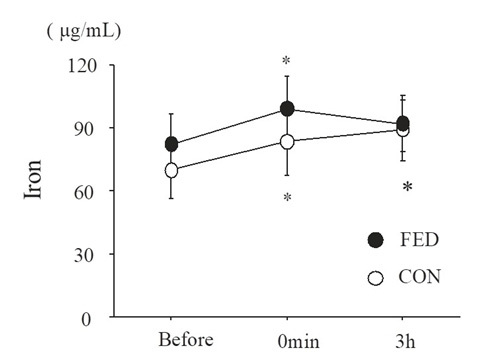
Serum iron levels before and after exercise. Values are means ± SE. *; P < 0.05 vs. before.

Fig. 3 presents serum iron levels before and after exercise. Serum iron levels significantly increased immediately after exercise (main effect for time, *P* < 0.01). However, no significant differences were observed between the FED (98.5 ± 15.8 µg/mL) and CON (82.9 ± 15.3 µg/mL) conditions at any time point (main effect for condition, *P* = 0.475). 

**Figure 4.**
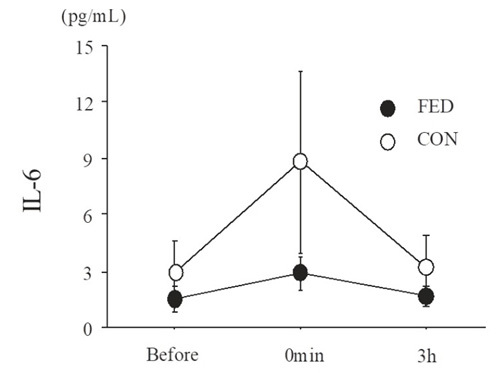
Plasma interleukin-6 (IL-6) levels before and after exercise. Values are means ± SE.

Fig. 4 presents plasma IL-6 levels before and after exercise. Plasma IL-6 levels did not significantly change after exercise under either condition (main effect for time, *P* = 0.153). Moreover, no significant differences were detected between the conditions at any time point (main effect for condition, *P *= 0.168). 

**Figure 5.**
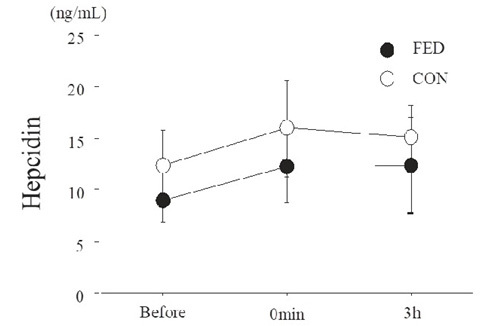
Serum hepcidin levels before and after exercise. Values are means ± SE.

Fig. 5 presents serum hepcidin levels before and after exercise. Serum hepcidin levels did not significantly change after exercise in either condition (main effect for time, *P* = 0.118). Moreover, no significant differences were observed between conditions at any time point (main effect for condition, *P* = 0.295).

## DISCUSSION

The present study determined the effects of consuming a meal before endurance exercise on post-exercise increase in serum hepcidin levels in untrained, young females. The main finding was that the pre-exercise meal did not affect exercise-induced hepcidin response after exercise. Moreover, serum hepcidin levels did not change significantly during the 3 h post-exercise period under either the FED or CON conditions. Lower absolute exercise intensity might be the reason for the lack of an increase in hepcidin after exercise. 

HR and RPE during exercise did not significantly differ between the FED and CON conditions. In addition, energy expenditure during exercise was not significantly different between the two conditions. Thus, 60 min of exercise under each condition was conducted under a similar physiological stimulus. However, RER (an indication of the substrate oxidation pattern) during exercise was significantly higher in the FED condition than that in the CON condition. These results indicate that carbohydrate utilization was enhanced during exercise in the FED condition, as previously reported^27^. Furthermore, blood glucose levels were significantly higher in the FED condition than those in the CON condition immediately after the completion of exercise. Blood lactate levels significantly increased immediately after exercise under both conditions; however, the exercise-induced elevation was significantly greater in the FED condition than that in the CON condition. Therefore, consuming a meal before endurance exercise promoted glycolysis, which caused higher blood lactate levels above the onset of blood lactate accumulation.

 Several studies have shown that IL-6 stimulates hepcidin secretion after exercise^6,13,14,28,29^. In the present study, plasma IL-6 and serum hepcidin levels did not significantly change after exercise under either condition, with no significant difference between conditions. Sim et al. (2013) reported that IL-6 and hepcidin levels significantly increased after 40 min of pedaling exercise at 65% of $\dot{V}$O_2max_ (the same relative intensity utilized in the present study)^30^. Therefore, it was assumed that the exercise intensity (65% of $\dot{V}$O_2max_) and duration (60 min) utilized in the present study would be adequate to stimulate IL-6 and hepcidin responses. However, it is plausible that the absolute intensity (93 ± 7 W) was insufficient to evoke IL-6 and hepcidin responses due to the lower fitness levels ($\dot{V}$O_2max_) in the present subjects (i.e., untrained females). Therefore, caution should be taken when interpreting our results and further determinations are required among different populations with higher $\dot{V}$O_2max_ values (e.g., physiologically active females).

 Serum iron levels significantly increased immediately after exercise under both conditions, in agreement with previous studies1^2-14,18-20,31^; exercise-induced hemolysis may be involved^32^. Hepcidin production is upregulated by elevated levels of iron in blood to maintain iron homeostasis^33^ and higher serum iron levels may stimulate hepcidin secretion^34^. Many studies have reported hepcidin responses after running^12-14,19^. However, running promotes hemolysis owing to mechanical effects of the sole of the foot. Telford et al. (2003) compared exercise-induced hemolysis between running and cycling at 75% of $\dot{V}$O_2max_ and reported lower hemolysis during cycling than running^5^. Therefore, the exercise modality utilized in the present study (cycling) might partially explain the lack of an exercise-induced increase in hepcidin under both conditions. 

 Serum ferritin levels before exercise did not significantly differ between the two conditions. None of the subjects developed anemia in the present study. However, 6 of the 10 subjects had lower serum ferritin levels (≤ 20 ng/mL) and these subjects were considered to be iron deficient^35-37^. Peeling et al. (2017) demonstrated a positive correlation between serum ferritin levels at baseline and exercise-induced increases in hepcidin levels. Post-exercise hepcidin elevation has been attenuated in individuals with iron deficiency^34^. Therefore, lower iron status with lower ferritin levels in the present subjects is related to the lack of change in serum hepcidin levels following exercise. 

The influence of the menstrual cycle on iron metabolism should be considered. Females with a normal menstrual cycle lose 20–80 mL blood, which is equivalent to a loss of 10–40 mg iron during menstruation^38,39^. We monitored individual menstrual cycles before the study, and the two conditions were undertaken during the follicular phase (7–11 d after the onset of menstruation) in different months. Serum hepcidin levels tended to be lower in females during the menstrual phase^40^. However, the effects of iron loss from menstruation appeared to be small because the two conditions exercised during the latter part of the follicular phase (i.e., after completing menstruation). Moreover, Jilma et al. (1997) determined the effects of the menstrual cycle on IL-6 concentrations in 18 premenopausal females^41^. These authors showed that IL-6 did not significantly differ during the different menstrual phases. Based on these findings, the influence of the menstrual cycle on the present results (e.g., IL-6 and hepcidin responses) was considered minor. 

As a limitation of the present study, we were unable to conduct a dietary survey (e.g., regarding to total energy intake and iron intake). Increasing iron intake augments basal serum hepcidin levels in female long-distance runners^42^. Therefore, further studies to address the effects of daily energy intake on the exercise-induced increases in hepcidin may be important. 

In conclusion, serum hepcidin and plasma IL-6 levels were not significantly affected by 60 min of endurance exercise despite the consumption of a meal before exercise. In addition, contrary to our hypothesis, 60 min of endurance exercise at 65% of $\dot{V}$O_2max_ did not increase hepcidin or IL-6 levels under either fed or fasted conditions. From a practical viewpoint, the findings in the present study suggest that skipping meals before endurance exercise would not alter exercise-induced hepcidin responses in untrained young females. Therefore, for sedentary young females, greater attention to dietary iron intake is important for preventing iron deficiency.
